# Numerical simulation for peristalsis of Carreau-Yasuda nanofluid in curved channel with mixed convection and porous space

**DOI:** 10.1371/journal.pone.0170029

**Published:** 2017-02-02

**Authors:** Anum Tanveer, T. Hayat, A. Alsaedi, B. Ahmad

**Affiliations:** 1 Department of Mathematics, Quaid-I-Azam University 45320, Islamabad 44000, Pakistan; 2 Nonlinear Analysis and Applied Mathematics (NAAM) Research Group, Department of Mathematics, Faculty of Science, King Abdulaziz University, Jeddah 21589, Saudi Arabia; North China Electric Power University, CHINA

## Abstract

Main theme of present investigation is to model and analyze the peristaltic activity of Carraeu-Yasuda nanofluid saturating porous space in a curved channel. Unlike the traditional approach, the porous medium effects are characterized by employing modified Darcy’s law for Carreau-Yasuda fluid. To our knowledge this is first attempt in this direction for Carreau-Yasuda fluid. Heat and mass transfer are further considered. Simultaneous effects of heat and mass transfer are examined in presence of mixed convection, viscous dissipation and thermal radiation. The compliant characteristics for channel walls are taken into account. The resulting complex mathematical system has been discussed for small Reynolds number and large wavelength concepts. Numerical approximation to solutions are thus plotted in graphs and the physical description is presented. It is concluded that larger porosity in a medium cause an enhancement in fluid velocity and reduction in concentration.

## 1 Introduction

Nanomaterials are known to posses increase in heat transfer processes like waste heat recovery, automobile radiators, thermal management, heat exchangers and refrigeration. The colloidal suspension of nanometer sized particles (metallic or non-metallic) in traditional fluids give rise to nanofluids. Such fluids with an improvement in thermal conductivity and thermal diffusivity enhance the heat transfer of conventional fluids. Further involvement of nanofluids in heat transfer process reduces the capital costs and upgrade the energy conversion and efficiency. The preparation of nanofluids is due to addition of materials like metals, non-metals, carbides and hybrid etc into water, oil or glycols. Out of existing models of nanofluids the Buongiorno [1] model emphasizes that heat transfer is mainly due to thermophoresis and Brownian diffusion. Since then extensive literature is available on the topic (see refs. [2–11]).

Occurrence of peristalsis (involuntary contractions and relaxations) is of fundamental importance in human physiology and modern industry. The physiologists are familiar with peristalsis since its involvement in digestive and reproductive tract of human beings. However research on the topic is initiated by Latham [12] and Shapiro et al. [13]. At present pumping machinery functions through principle of peristalsis. Some examples here include roller, finger and hose pumps, domestic waste management pumps, dialysis machines, oxygenation and so forth. Up till now the discussion on peristalsis for planer channel in existing literature is extensive (see refs. [14–18]). It is important to note that most of the physical systems and human arteries are naturally curved in shape. However perhaps due to complex mathematical description, the curved channel flows are less focused by the researchers (see refs. [19–23]). Further CY- fluid receives special attention since it interpolates between zero-shear-rate viscosity (Newtonian behavior) and the infinite-shear rate viscosity (non-Newtonian behavior). The involvement of two parameters (*n*) exhibits the degree of shear-thinning and the size and curvature of medium between Newtonian and shear-thinning behavior of CY- fluid. No doubt the literature available on peristalsis of Carreau-Yasuda fluid is countable (see refs. [24–26]).

Many applications in geophysical and industrial engineering involve conjugate phenomenon of the heat and mass transfer which occurs as a consequence of buoyancy effects. The simultaneous effects of heat and mass transfer are found handy in the improvement of energy transport technologies, metallurgy, power generation, production of polymers and ceramics, food drying, oil recovery, food processing, fog dispersion, the distribution of temperature and moisture in the field of agriculture and so-forth. Some relevant studies can be consulted via refs. [27–33]. The combination of heat and mass transfer effects in porous media found its utility in engineering and geophysical area such as in geothermal reservoirs, drying of porous solids, thermal insulation, catalytic reactors, nuclear reactor coolers and underground energy transport. Few attempts in this direction can be mentioned by the studies [34, 35].

The flow execution in natural and artificial environmental systems (like petroleum reservoirs, composites manufacture process, water flow on the ground, chemical reactors, filters, circulation of capillaries) is through the porous space. The qualitative description of flow saturated in porous space dates back to the experimental work of Darcy [36]. Darcy’s observations are then exploited to obtain several mathematical models for fluid flow comprising porous media. Up till now no effort has been made to explore the peristaltic fluid flow in curved channel with porous medium followed by modified Darcy’s law. The problem in hand is one such attempt. Flow stream is developed for Carreau-Yasuda nanofluid in a curved channel. Effectiveness of buoyancy is executed through mixed convection. Further thermal radiation and viscous dissipation effects are also present. The graphical interpretation is made through numerical solutions. The physical significance of involved parameters is pointed out in the last section. In addition nomenclature of the involved parameters has been provided in Table 1.

**Table 1:** 

Parameters with units	σ* Stefan-Boltzmann constant (Wm/K^4^)
**V** velocity (m/s)	$\bar{a}$ amplitude of wave (m)
$\bar{v}$, $\bar{u}$ radial and axial velocity components (m/s)	*β* material fluid parameters
$\bar{x}$, axial coordinate (m)	l* mean absorption coefficient (1/m)
$\bar{r}$ radial coordinate (m)	*T*_*m*_ mean fluid temperature (K)
$\bar{t}$ time (s)	τ* elastic tension (kg/s^2^)
*c* speed of wave (m/s)	*d*′ viscous damping coefficient (kg/m^2^/*s*)
$\bar{d}$ half channel width (m)	m_1_ mass per unit area (kg/m^2^)
*ρ*_*p*_ density of nanoparticles (kgm^−3^)	Dimensionless parameters
$\pm \bar{\eta }$ displacement of walls (m)	*ψ* stream function
*μ*_0_ dynamic viscosity (kg/ms)	*θ*, *ϕ* dimensionless temperature & concentration
*υ* kinematic viscosity (m^2^ s^−1^)	Z heat transfer rate
*ρ*_*f*_ fluid density (kgm^−3^)	Qr, Gr Grashof numbers
*c*_*p*_ Specific heat (m^2^ s^−2^)	*ϵ* amplitude ratio parameter
*k*_1_ thermal conductivity (WK^−1^ m^−1^)	Rd radiation parameter
**S** Extra stress tensor (kg/ms^2^)	Br Brinkman number
*S*_*ij*_ (i = 1,2, j = 1,2) stress components	Re Reynold number
*p* pressure (N/m^2^)	*δ* wave number
*β*_*C*_ concentration expansion coefficient	*We* Weissenberg number
*g* gravitational acceleration (ms^−2^)	*Sc* Schmidt number
*a*, n fluid parameters	*τ* ratio of heat capacity
*β*_*T*_ thermal expansion coefficient (1/K)	Pr Prandtl number
R* inner radius of curved channel (m)	*k* curvature parameter
*T*, *C* temperature (K) & concentration	*Ec* Eckert number
*T*_0_, *T*_1_ temperatures at the walls (K)	*E*_1_, *E*_2_, *E*_3_ elasticity parameters
*C*_0_, *C*_1_ concentration at the walls	Nt, thermophoresis diffusion coefficient
*D*_*B*_ Brownian diffusion coefficient (m^2^/*s*)	Nb Brownian diffusion coefficients
*D*_*T*_ Thermophoretic diffusion coefficient (m^2^/*s*)	*β* viscosity ratio
$R_{\bar{r}}$, $R_{\bar{x}}$ porosity components (kg/m^2^ s^2^)	*a*, *n* fluid parameters
λ wavelength (m)	

## 2 Problems development

**Fig 1:**
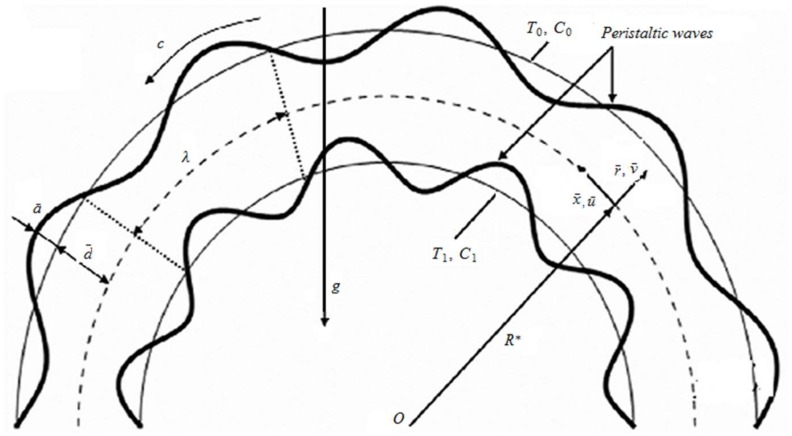
Geometry of the problem.

The mathematical modeling for an incompressible Carreau-Yasuda nanofluid in a channel configured in a circle of inner radius R* and separation $2\bar{d}$ is made in this section. The presence of porous medium between the curved walls of the channel is considered. The gravitational effects are taken into account. Here $\bar{r}$ signifies the radial-direction whereas $\bar{x}$ denotes the axial direction. The dynamics of fluid inside the channel boundaries is developed through the propagation of peristaltic waves along the channel walls (see Fig 1). Moreover relative to arterial like flow peristalsis the influential aspect of compliance in terms of wall’s stiffness, elasticity and damping is not ignored. The relative positions of the curved channel walls in radial direction can be visualized through the following expression:

$$\bar{r}=\pm \bar{\eta }\left(\bar{x},\bar{t}\right)=\pm \left[\bar{d}+\bar{a}\sin\frac{2\pi }{\lambda }\left(\bar{x}-c\bar{t}\right)\right],$$ (1)

where *c*, $\bar{a},$ λ denote the peristaltic wave speed, amplitude and length, $\bar{t}$ and $\pm \bar{\eta }$ the time and displacements of channel walls.

The problem under consideration can be put in mathematical form via conservation principles of mass, momentum, energy and nanoparticle volume fraction respectively. Thus following the procedure of [3, 4, 6] one obtains

Continuity equation

$$\frac{\partial \bar{v}}{\partial \bar{r}}+\frac{R^{*}}{\bar{r}+R^{*}}\frac{\partial \bar{u}}{\partial \bar{x}}+\frac{\bar{v}}{\bar{r}+R^{*}}=0,$$ (2)

$\bar{r}$-component of momentum equation:

$$\begin{array}{ccc} \rho_{f}\left[\frac{d\bar{v}}{d\bar{t}}-\frac{{\bar{u}}^{2}}{\bar{r}+R^{*}}\right] & = & -\frac{\partial \bar{p}}{\partial \bar{r}}+\frac{1}{\bar{r}+R^{*}}\frac{\partial }{\partial \bar{r}}\left\{\left(\bar{r}+R^{*}\right){\bar{S}}_{\bar{r}\bar{r}}\right\}\\ & & +\frac{R^{*}}{\bar{r}+R^{*}}\frac{\partial {\bar{S}}_{\bar{x}\bar{r}}}{\partial \bar{x}}-\frac{{\bar{S}}_{\bar{x}\bar{x}}}{\bar{r}+R^{*}}+R_{\bar{r}}, \end{array}$$ (3)

*x*-component of momentum equation:

$$\begin{array}{ccc} \rho_{f}\left[\frac{d\bar{u}}{d\bar{t}}+\frac{\bar{u}\bar{v}}{\bar{r}+R^{*}}\right] & = & -\frac{R^{*}}{\bar{r}+R^{*}}\frac{\partial \bar{p}}{\partial \bar{x}}+\frac{1}{{\left(\bar{r}+R^{*}\right)}^{2}}\frac{\partial }{\partial \bar{r}}\left\{{\left(\bar{r}+R^{*}\right)}^{2}{\bar{S}}_{\bar{r}\bar{x}}\right\}+\frac{R^{*}}{\bar{r}+R^{*}}\frac{\partial {\bar{S}}_{\bar{x}\bar{x}}}{\partial \bar{x}}\\ & & +g\rho_{f}\beta_{T}\left(T-T_{0}\right)+\left(\rho_{p}-\rho_{f}\right)g\beta_{C}\left(C-C_{0}\right)+R_{\bar{x}}. \end{array}$$ (4)

Energy equation:

$$\begin{array}{ccc} {\left(\rho c\right)}_{p}\left[\frac{dT}{d\bar{t}}\right] & = & k_{1}\nabla^{2}T+\tau {\left(\rho c\right)}_{p}D_{B}\left(\nabla C.\nabla T\right)+\frac{\tau {\left(\rho c\right)}_{p}D_{T}}{T_{m}}\left(\nabla T.\nabla T\right)\\ & & -\frac{\partial }{\partial \bar{r}}\left(\frac{-16\sigma^{*}T_{0}^{3}}{3l^{*}}\frac{\partial T}{\partial \bar{r}}\right)+\tau .L, \end{array}$$ (5)

Nanoparticles mass transfer equation:

$$\frac{dC}{d\bar{t}}=D_{B}\left(\nabla^{2}C\right)+\frac{D_{T}}{T_{m}}\left(\nabla^{2}T\right).$$ (6)

The Cauchy stress tensor *τ* and extra stress tensor $\bar{S}$ for Carreau–Yasuda fluid model are [25]:

$$\tau =-\bar{p}I+\bar{S},$$ (7)

$$\bar{S}=\mu \left(\overset{´}{\gamma }\right)A_{1},$$ (8)

in which **A**_1_ represents the first Rivlin-Erickson tensor and the apparent viscosity $\mu (\overset{´}{\gamma })$ can be obtained through the following relation:

$$\mu \left(\overset{´}{\gamma }\right)=\mu_{\infty }+\left(\mu_{0}-\mu_{\infty }\right){\left[1+{\left(\Gamma \overset{´}{\gamma }\right)}^{a}\right]}^{\frac{n-1}{a}},$$ (9)

where $\overset{´}{\gamma }=\sqrt{2t\bar{r}\left(D^{2}\right)}$ and $D=\frac{1}{2}\left[\text{grad}V+\text{grad}V^{T}\right].$ The involvement of zero and infinite shear-rate viscosities *μ*_0_ and *μ*_∞_ and the Carreau–Yasuda fluid parameters *a*, *n* and Γ provide an edge to this fluid model to the associated characteristics of these five quantities. Firstly in the range of high shear rate the dominance of viscous effects can be defined by *μ*_0_ and *μ*_∞_ along the channel walls. On the other hand the shear thinning/thickening behavior can be predicted through the parameters *a*, *n* and Γ. Actually the functioning of asymptotic viscosities (*μ*_0_ and *μ*_∞_) is responsible for fluid regulation in the non-Newtonian arrangement. Moreover in Carreau-Yasuda fluid model the specific values of parameters can form the numerous concentrated polymer solutions such as *a* = 2 and *μ*_∞_ = 0. Fixed value of Yasuda parameter *a* = 2 represents the Carreau model. The value of Yasuda parameter is fixed in this problem at *a* = 1. Also

$$\nabla^{2}={\left(\frac{R^{*}}{\bar{r}+R^{*}}\right)}^{2}\frac{\partial^{2}}{\partial {\bar{x}}^{2}}+\frac{1}{\bar{r}+R^{*}}\frac{\partial }{\partial \bar{r}}\left\{(\bar{r}+R^{*})\right\}.$$

The quantities appearing in above equations represent the velocity $V=(\bar{v}\left(\bar{r},\bar{x},\bar{t}\right),\bar{u}\left(\bar{r},\bar{x},\bar{t}\right),0)$ in radial and axial directions respectively, the material derivative in curved channel $\frac{d}{d\bar{t}}=\frac{\partial }{\partial \bar{t}}+\bar{v}\frac{\partial }{\partial \bar{r}}+\frac{{\bar{r}}^{*}\bar{u}}{\bar{r}+{\bar{r}}^{*}}\frac{\partial }{\partial \bar{x}}$, the heat capacity to fluid’s material ratio $\tau =\frac{{\left(\rho c\right)}_{\bar{p}}}{{\left(\rho c\right)}_{f}}$, the kinematic viscosity of fluid *ν*, the specific heat *c*_*p*_, the pressure $\bar{p}$, the fluid density *ρ*_*f*_, the nanoparticle density *ρ*_*p*_, the gravitational acceleration *g*, the thermal expansion coefficient *β*_*T*_, the concentration expansion coefficient *β*_*C*_, the temperatures at the lower and upper channel walls *T*_0_, *T*_1_, the concentrations at the lower and upper channel walls *C*_0_, *C*_1_, the Brownian diffusion parameter *D*_*B*_, the thermophoresis diffusion parameter *D*_*T*_, the Stefan-Boltzmann constant σ*, the mean absorption coefficient l*, the mean temperature of fluid *T*_*m*_, the temperature and concentration of fluid *T* and *C*, the Darcy resistance in porous medium $R=(R_{\bar{r}},R_{x},0)$. The pressure drop and velocity are related by Darcy’s law. However for Carreau-Yasuda fluid the relation is followed by newly developed modified Darcy’s law preserving following filtration forms:

$$\nabla \bar{p}=\frac{-\xi }{\underline{\text{K}}}\left[\mu_{\infty }+\left(\mu_{0}-\mu_{\infty }\right){\left[1+{\left(\Gamma \overset{´}{\gamma }\right)}^{a}\right]}^{\frac{n-1}{a}}\right]V,$$ (10)

where the permeability and porosity of porous medium are represented by Ḵ and *ξ* respectively. The above generalized form is capable of recovering the results of Darcy law for large *a* (*a* → ∞) or by assuming *n* = 1. Since flow resistance containing porous space can be explained in terms of pressure gradient, thus Eq (10) can be written as:

$$R=\frac{-\xi }{\underline{\text{K}}}\left[\mu_{\infty }+\left(\mu_{0}-\mu_{\infty }\right){\left[1+{\left(\Gamma \overset{´}{\gamma }\right)}^{a}\right]}^{\frac{n-1}{a}}\right]V.$$ (11)

The extra stress components ${\bar{S}}_{\hat{x}\bar{r}},{\bar{S}}_{\bar{r}\bar{r}}$ and ${\bar{S}}_{\hat{x}\hat{x}}$ of $\bar{S}$ in Carreau-Yasuda fluid can be obtained using Eq (8). It is remarkable to mention that the Rosseland approximation corresponding to radiative heat flux is utilized in Eq (5) to obtain the relevant radiation term. In considered problem, the no-slip condition, prescribed surface temperature and concentration values at the channel boundaries and the compliant properties of wall can be put in the following forms:

$$\begin{array}{ccc} \bar{u} & = & 0,\;\;\text{at}\;\;\bar{r}=\pm \bar{\eta }, \end{array}$$ (12)

$$\begin{array}{ccc} T & = & \left\{\begin{array}{c} T_{1}\\ T_{0} \end{array}\right\}\text{,}\;\;\;\;\;\text{at}\;\;\bar{r}=\pm \bar{\eta }, \end{array}$$ (13)

$$\begin{array}{ccc} C & = & \left\{\begin{array}{c} C_{1}\\ C_{0} \end{array}\right\}\text{,}\;\;\;\;\;\text{at}\;\;\bar{r}=\pm \bar{\eta }, \end{array}$$ (14)

$$\begin{array}{ccc} & & \left.\frac{R^{*}}{\bar{r}+R^{*}}\left[-\tau^{*}\frac{\partial^{3}}{\partial {\bar{x}}^{3}}+m_{1}\frac{\partial^{3}}{\partial \bar{x}\partial {\bar{t}}^{2}}+d^{\prime }\frac{\partial^{2}}{\partial \bar{t}\partial \bar{x}}\right]\bar{\eta }=-\rho_{f}\left[\frac{d\bar{u}}{d\bar{t}}+\frac{\bar{u}\bar{v}}{\bar{r}+R^{*}}\right]+\frac{1}{{\left(\bar{r}+R^{*}\right)}^{2}}\frac{\partial }{\partial \bar{r}}\left\{{\left(\bar{r}+R^{*}\right)}^{2}{\bar{S}}_{\bar{r}\bar{x}}\right\}+\right.\\ & & \left.\frac{R^{*}}{\bar{r}+R^{*}}\frac{\partial {\bar{S}}_{\bar{x}\bar{x}}}{\partial \bar{x}}+g\rho_{f}\beta_{T}\left(T-T_{0}\right)+\left(\rho_{p}-\rho_{f}\right)g\beta_{C}\left(C-C_{0}\right)+R_{\bar{x}},\;\;\;\text{at}\;\bar{r}=\pm \bar{\eta }\right., \end{array}$$ (15)

where τ*, *m*_1_, *d*′ exhibit the coefficients of elastic tension in the membrane, mass per unit area and viscous damping respectively.

Consideration of non-dimensional quantities and stream function $\bar{\psi }(\bar{r},\bar{x},\bar{t})$ by the definitions below will lead to required set of equations as follows:

$$\begin{array}{ccc} \psi & = & \frac{\bar{\psi }}{c\bar{d}},\;x=\frac{\bar{x}}{\lambda },\;r=\frac{\bar{r}}{\bar{d}},\;t=\frac{c\bar{t}}{\lambda },\;\eta =\frac{\bar{\eta }}{\bar{d}},\;k=\frac{R^{*}}{\bar{d}},\\ \theta & = & \frac{T-T_{0}}{T_{1}-T_{0}},\;\phi =\frac{C-C_{0}}{C_{1}-C_{0}},\;S_{ij}=\frac{\bar{d}}{\mu c}{\bar{S}}_{ij},\;p=\frac{\bar{p}{\bar{d}}^{2}}{c\lambda \mu },\;\epsilon =\frac{\bar{a}}{\bar{d}},\\ Gr & = & \frac{\rho_{f}g\beta_{T}{\bar{d}}^{2}\left(T_{1}-T_{0}\right)}{c\mu },\;Qr=\frac{\left(C_{1}-C_{0}\right)\left(\rho_{p}-\rho_{f}\right)g\beta_{C}{\bar{d}}^{2}}{c\mu },\\ \text{Re} & = & \frac{\rho_{f}c\bar{d}}{\mu },\;Nb=\frac{\tau D_{B}\left(C_{1}-C_{0}\right)}{\upsilon },\;Nt=\frac{\tau \rho_{p}D_{T}\left(T_{1}-T_{0}\right)}{\upsilon T_{m}},\mathrm{Pr}=\frac{\mu c_{f}}{k_{1}},\\ Ec & = & \frac{c^{2}}{c_{p}\left(T_{1}-T_{0}\right)},\;Br=Ec\mathrm{Pr},\;Rd=\frac{16\sigma^{*}T_{0}^{3}}{3k^{*}\mu c_{f}},\;\delta =\frac{\bar{d}}{\lambda },\;Sc=\frac{D_{B}}{\upsilon },\;\\ E_{1} & = & -\frac{\tau^{*}{\bar{d}}^{3}}{\lambda^{3}\mu c},\;E_{2}=\frac{m_{1}c{\bar{d}}^{3}}{\lambda^{3}\mu },\;E_{3}=\frac{{\bar{d}}^{3}d^{\prime }}{\mu \lambda^{2}},\;u=\frac{\bar{u}}{c},\;v=\frac{\bar{v}}{c},\;Da=\frac{\underline{\text{K}}}{{\bar{d}}^{2}\xi }, \end{array}$$ (16)

$$u=-\frac{\partial \psi }{\partial r},\;\;\;\;v=\frac{\delta k}{r+k}\frac{\partial \psi }{\partial x},$$

in which the non-dimensional quantites above are the definitions of following physical parameters: *δ* the wave number, *ϵ* the amplitude ratio parameter, Re the Reynolds number, Pr the Prandtl number, *E*_1_, *E*_2_, *E*_3_ the elasticity parameters, *Rd* the radiation parameter, *Gr* the local temperature Grashof number, *Qr* the local nanoparticles Grashof number, *Ec* the Eckert number, *Br* the Brinkman number, *Nt*, *Nb* the thermophoresis and Brownian motion parameters respectively, *Sc* the Schmidt number and *Da* the Darcy number.

Thus utilization of above parameters and long wavelength approximation yield:

$$\frac{\partial p}{\partial r}=0,$$ (17)

$$\begin{array}{ccc} & & \left.\left.\frac{-k}{r+k}\frac{\partial p}{\partial x}+\frac{1}{{\left(r+k\right)}^{2}}\frac{\partial }{\partial r}\left[{\left(r+k\right)}^{2}S_{rx}\right]\right.+\left(Gr\theta +Qr\phi \right)\right.\\ & & \left.+\frac{1}{Da}\frac{\partial \psi }{\partial r}\left[1+We\left(1-\beta \right)\left(n-1\right)\left(-\frac{\partial^{2}\psi }{\partial r^{2}}+\frac{1}{r+k}\frac{\partial \psi }{\partial r}\right)\right]=0,\right. \end{array}$$ (18)

$$\begin{array}{ccc} & & \left.\left.\frac{\partial^{2}\theta }{\partial r^{2}}+\frac{1}{r+k}\frac{\partial \theta }{\partial \bar{r}}+Br{\left(-\frac{\partial^{2}\psi }{\partial r^{2}}+\frac{1}{r+k}\frac{\partial \psi }{\partial r}\right)}^{2}\left[1+We\left(1-\beta \right)\left(n-1\right)\left(-\frac{\partial^{2}\psi }{\partial r^{2}}+\frac{1}{r+k}\frac{\partial \psi }{\partial r}\right)\right]\right.\right.\\ & & \left.+\mathrm{Pr}Rd\left(\frac{\partial^{2}\theta }{\partial r^{2}}\right)+\mathrm{Pr}Nt\left(\frac{\partial^{2}\theta }{\partial r^{2}}\right)+\mathrm{Pr}Nb\left(\frac{\partial \theta }{\partial r}\frac{\partial \phi }{\partial r}\right)=0,\right. \end{array}$$ (19)

$$\left(\frac{\partial^{2}\phi }{\partial r^{2}}+\frac{1}{r+k}\frac{\partial \phi }{\partial r}\right)+\frac{Nt}{Nb}\left(\frac{\partial^{2}\theta }{\partial r^{2}}+\frac{1}{r+k}\frac{\partial \theta }{\partial \bar{r}}\right)=0,$$ (20)

$$\eta =1+\epsilon \sin2\pi \left(x-t\right),$$ (21)

$$\frac{\partial \psi }{\partial r}=0\;\;\text{at}\;\;r=\pm \eta ,$$ (22)

$$\theta =\left\{\begin{array}{c} 1\\ 0 \end{array}\right\}\;\;\text{at}\;\;\;\;\;r=\pm \eta ,$$ (23)

$$\phi =\left\{\begin{array}{c} 1\\ 0 \end{array}\right\}\;\;\text{at}\;\;\;\;\;r=\pm \eta ,$$ (24)

$$\begin{array}{ccc} & & \frac{k}{r+k}\left[E_{1}\frac{\partial^{3}}{\partial x^{3}}+E_{2}\frac{\partial^{3}}{\partial x\partial t^{2}}+E_{3}\frac{\partial^{2}}{\partial t\partial x}\right]\bar{\eta }=\frac{1}{{\left(r+k\right)}^{2}}\frac{\partial }{\partial r}\left[{\left(r+k\right)}^{2}S_{rx}\right]\\ & & +\left.\left(Gr\theta +Qr\phi \right)+\frac{1}{Da}\frac{\partial \psi }{\partial r}\left[1+We\left(1-\beta \right)\left(n-1\right)\left(-\frac{\partial^{2}\psi }{\partial r^{2}}+\frac{1}{r+k}\frac{\partial \psi }{\partial r}\right)\right],\;\text{at}\;r=\pm \eta \right., \end{array}$$ (25)

The equation of stream function can be obtained from Eqs (17) and (18) by eliminating pressure. Thus one gets

$$\frac{\partial }{\partial r}\left[\frac{1}{\left(r+k\right)}\frac{\partial }{\partial r}\left[{\left(r+k\right)}^{2}S_{rx}\right]+\left(r+k\right)\left(Gr\theta +Qr\phi \right)\right]$$ (26)

$$+\frac{\partial }{\partial r}\left[\frac{1}{Da}\frac{\partial \psi }{\partial r}\left\{1+We\left(1-\beta \right)\left(n-1\right)\left(-\frac{\partial^{2}\psi }{\partial r^{2}}+\frac{1}{r+k}\frac{\partial \psi }{\partial r}\right)\right\}\right]=0,$$ (27)

where

$$S_{rx}=\left(-\frac{\partial^{2}\psi }{\partial r^{2}}+\frac{1}{r+k}\frac{\partial \psi }{\partial r}\right)\left[1+We^{a}\frac{\left(1-\beta )(n-1\right)}{a}{\left(-\frac{\partial^{2}\psi }{\partial r^{2}}+\frac{1}{r+k}\frac{\partial \psi }{\partial r}\right)}^{a}\right].$$ (28)

Here $\beta =\frac{\mu_{\infty }}{\mu_{0}}$ and $We=\frac{\Gamma c}{d}$ depict the viscosity ratio parameter and Weissenberg number respectively. It can be verified that for *n* = 1 or *We* = 0 the results of the viscous nanofluid with porous medium can be recorded as a special case of present problem. Heat transfer rate *Z* at the channel boundary can be obtained through the involvement of temperature as follows:

$$Z=\frac{\partial \eta }{\partial x}{\left|\frac{\partial \theta }{\partial r}\right|}_{r=\eta }.$$ (29)

## 3 Numerical method

The above mentioned problem results in the non-linear coupled system of equations whose explicit solution seems difficult to attain. However with the intense algorithmic advancement many built-in solution softwares are available at present. *Mathematica* is one of these. The exact as well as numerical approximation to solution expressions can be obtained efficiently through mathematica. *Mathematica* built-in routine NDSolve provides level of numerical computation with its systematic algorithm selection, automatic error tracking and precision arithmetics. Here we solve the above system numerically to skip the complexity of solutions and to obtain the graphical results directly. Thus the graphical description of pertinent parameters towards axial velocity *u*, temperature *θ*, concentration *ϕ* and heat transfer coefficient *Z* has been made in this section. Particularly the development of *u*, *θ*, *ϕ* and *Z* with the varying values of heat and mass transfer Grashof numbers *Gr* and *Qr*, thermophoresis and Brownian motion parameters *Nt* and *Nb*, wall compliant parameters *E*_1_, *E*_2_, *E*_3_, Darcy number *Da*, viscosity ratio parameter *β*, fluid parameter *n*, curvature parameter *k*, Prandtl number *Pr*, Brinkman number *Br*, radiation parameter *Rd*, Weissenberg number *We* will be emphasized via physical basis.

### 3.1 Velocity distribution

**Fig 2:**
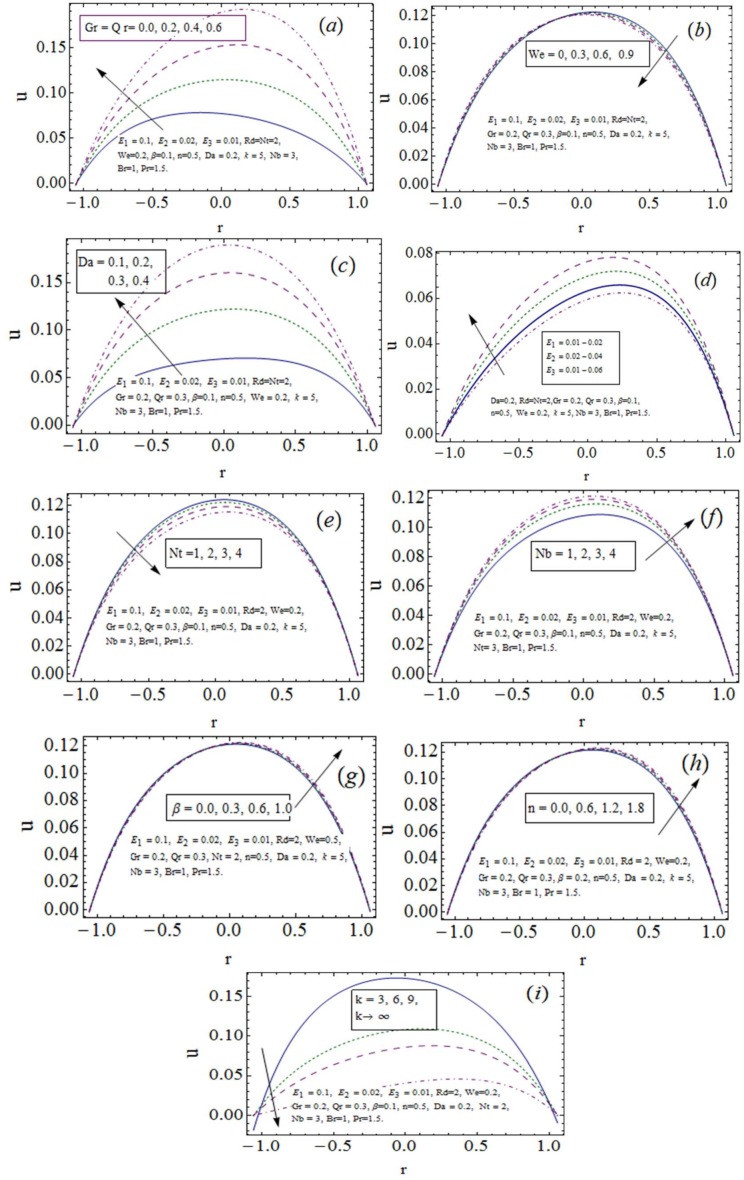
Axial velocity *u* variation with *x* = 0.2, *t* = 0.1, *ϵ* = 0.1.

Developments in velocity distribution as a result of variation in different embedding parameters are sketched in this subsection via Fig 2(*a*)–2(*i*). The axial velocity is noticed an increasing function of mixed convection parameters (Grashof numbers). It is due to viscosity drop (see Fig 2(*a*)). Mixed convection is proficient to provide energy dissipation in nuclear reactor technology and electronic cooling processes where forced convection fails to achieve required target. The dual response of *We* on velocity is captured in Fig 2(*b*). The porosity shows an increase in velocity since adding more pores causes flow easier in a medium. Thus increasing behavior of *u* is drawn through Fig 2(*c*). Clinically pores in walls of blood capillaries allow exchange of water, oxygen and many other nutrients between the blood and the tissues. Growing values of wall elastic parameters produce velocity development where damping effects oppositely (see Fig 2(*d*)). The results are found well matched with study [25]. The thermophoresis (*Nt*) lowers speed of nanoparticles that in turn lowers fluid velocity (see Fig 2(*e*)). On the other hand viscosity gets weak with Brownian diffusion (*Nb*) and so activation of *u* is observed with *Nb* (see Fig 2(*f*)). The results are compared with numerical studies [3, 6] for asymmetric and symmetric channels. The results of (Fig 2(*g*) & 2(*h*)) show dual response of *β* and *n* on *u*. It is seen that non-symmetric velocity rises near positive side of channel and it reduces near negative side. The decline in velocity with an increase in *k* is depicted in Fig 2(*i*). Due to curved flow configuration the velocity preserves non-symmetric behavior. Also *u* becomes flatten as straight channel is obtained (*k* → ∞).

### 3.2 Temperature distribution

**Fig 3:**
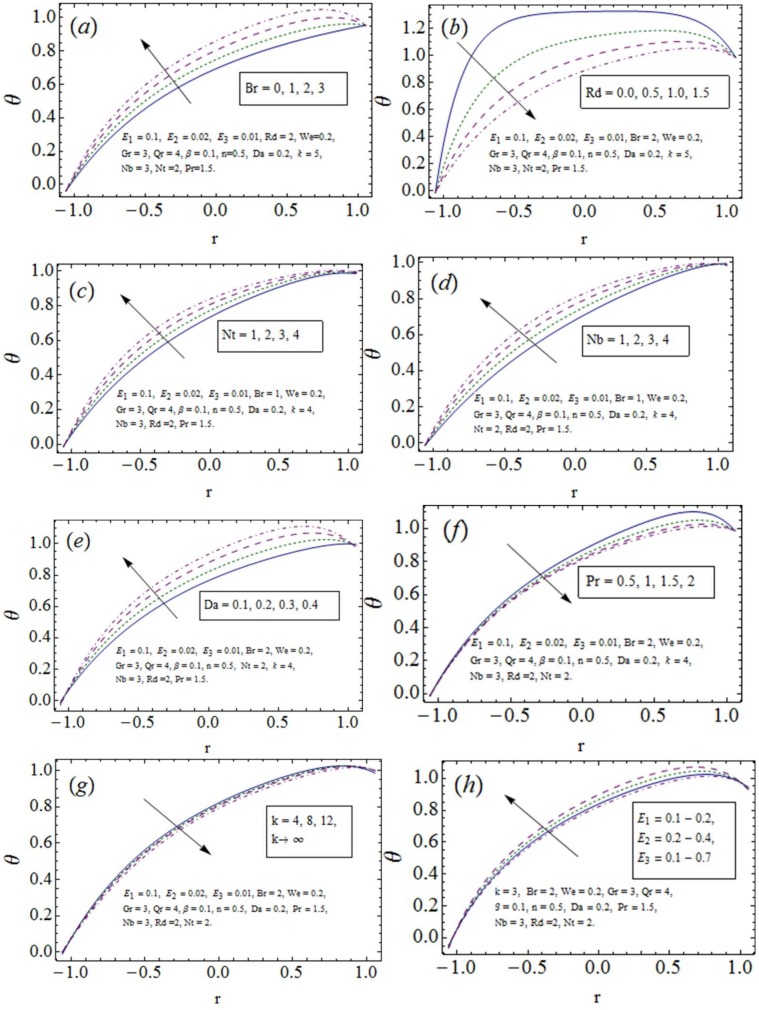
Temperature *θ* variation with *x* = 0.2, *x* = 0.1, *ϵ* = 0.1.

The physical description of embedded parameters on temperature *θ* is made in this subsection (see Fig 3(*a*)–3(*h*)). Impression of *Br* towards *θ* (see Fig 3(*a*)). Radiation indicates heat decay and thus decreasing response with an increase in *Rd* towards *θ* is noticed from Fig 3(*c*). An increase in *Nt* and *Nb* activates energy production and thus temperature rises (see (Fig 3(*c*) & 3(*d*)). Verification of results can be made with the study [6]. Increasing porosity (*Da*) causes rise in temperature since addition of pores causes growth in velocity and hence temperature of nanofluid (see Fig 3(*e*)). Since thermal conductivity decreases with an increase in *Pr* therefore decay in *θ* is noticed from Fig 3(*f*). The curvature tends to reduce the temperature when one moves from curved to planer channel (small to large *k*). Additionally greater impact is seen in case of curved channel (see Fig 3(*g*)). The wall elastic parameters *E*_1_ and *E*_2_ produce temperature development while temperature decays for *E*_3_ (see Fig 3(*h*)). Similar results have been reported by Hayat et al. [6, 16] via perturbation and numerical approaches respectively.

### 3.3 Nanoparticle volume fraction distribution

**Fig 4:**
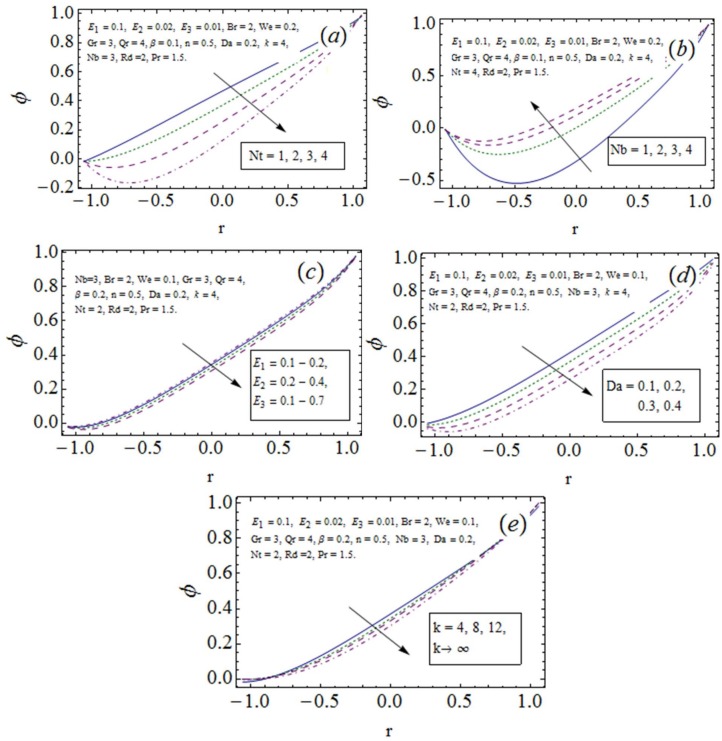
Nanoparticle mass transfer *ϕ* variation with *x* = 0.2, *t* = 0.1, *ϵ* = 0.1.

Fig 4(*a*)–4(*e*) communicate the development in nanoparticle volume fraction distribution *ϕ*. Decrease in *ϕ* is noticed with *Nt*. Hence there is diffusion enhancement with *Nt* (see Fig 4(*a*)). The density of nanoparticles enhances with growth of Brownian diffusion. Increase in *ϕ* is captured for larger *Nb* in Fig 4(*b*). The characteristics of wall compliant parameters on *ϕ* are found opposite from *u* and *θ* i.e., an increase in *E*_1_ and *E*_2_ correspond decline in *ϕ* where *E*_3_ causes promotion of *ϕ* [6, 16, 25]. Such results are anticipated since elasticity causes deformation of nutrients easier in case of blood veins and arteries where alternate effect of damping is recorded clinically (see Fig 4(c)). The decay of *ϕ* is noticed from Fig 4(*d*) for larger *Da*. Higher *Da* allow more pores in the medium which are responsible for diffusion of fluid and reduction of *ϕ*. The flow stream is converted to straight regime as we increase the value of curvature. From Fig 4(*e*) it is noticed that the volume fraction reduces when we move from curved to planer regimes (small to large *k*).

### 3.4 Heat transfer rate

**Fig 5:**
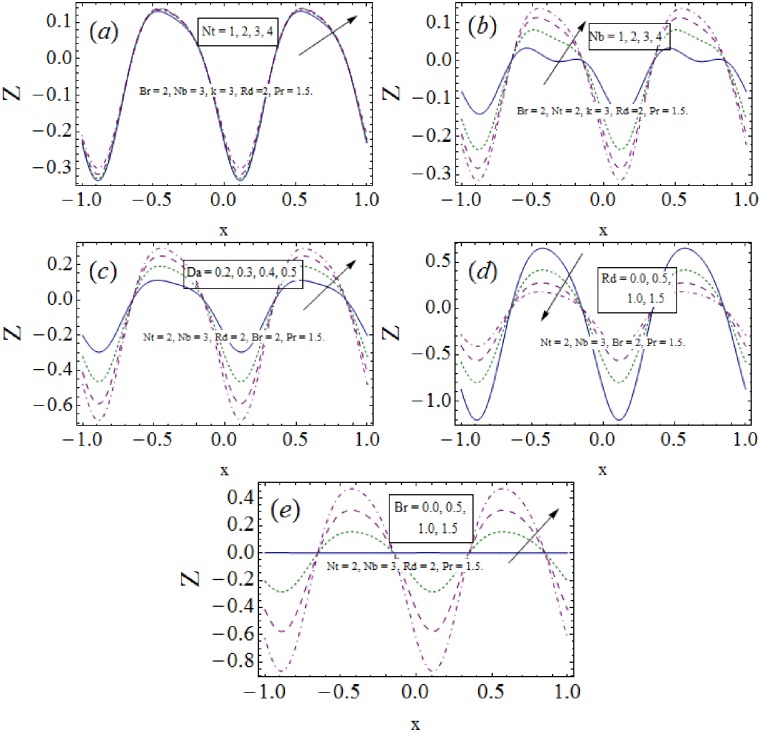
Heat transfer coefficient *Z* variation with *t* = 0.1, *ϵ* = 0.1.

The variation in absolute heat transfer rate *Z* under the influence of involved parameters is prepared in this subsection via Fig 5(*a*)–5(*e*). In favour of peristaltic waves along the channel boundaries the dual response of graphs towards *Z* is captured. The thermophoresis and Brownian diffusions (*Nt*, *Nb*) enhance the heat transfer rate (see (Fig 5(*a*) & 5(*b*)). The drawn results of Fig 5(*c*) indicate dominance of *Z* with higher values of *Da*. The results captured in (Fig 5(*d*) & 5(*f*)) have opposite responses of *Rd* and *Br* towards *Z* i.e., decline of *Z* is observed with rise in *Rd* whereas *Z* enhances for an increase in *Br*.

## 4 Conclusions

Mixed convection flow bounded in curved channel with compliant boundaries is developed for Carreau-Yasuda nanofluid. The observation is made for porous medium using modified Darcy’s law specifically. Such conditions are applicable in blood vessels where small pores allow exchange of water, ions, gases, lymph transport and other small molecules. An increase in porosity signifies disease states where endothelial barrier breaks down and allow large molecules like protein out of the vessel. In addition the thermal radiation and viscous dissipation effects are also examined. The particular points of this study are:

- The non-symmetric *u* is the outcome of curved channel.
- Mixed convection increases the fluid velocity.
- The fluid velocity, temperature and heat transfer rate show dominating behavior towards Darcy number where concentration falls for *Da*.
- The opposite results of *Nt* and *Nb* are seen for velocity and concentration.
- Weissenberg number preserves decelerating impact on velocity whereas fluid parameters *β* and *n* increase *u*.
- Reduction in *u*, *θ* and *ϕ* is noticed with an increase in curvature.
- Viscous dissipation affects *θ* and *Z* positively whereas alternative results of radiation are observed.
